# Generation of Customized Bone Implants from CT Scans Using FEA and AM

**DOI:** 10.3390/ma17174241

**Published:** 2024-08-27

**Authors:** Claude Wolf, Deborah Juchem, Anna Koster, Wilfrid Pilloy

**Affiliations:** 1 Department of Engineering, University of Luxembourg, 6 Rue Coudenhove-Kalergi, L-1359 Luxembourg, Luxembourg; 2 Department of Nuclear Medicine, Sefako Makgatho University, Ga-Rankuwa 0208, South Africa; medmaxtwo@gmail.com

**Keywords:** additive manufacturing, fused filament fabrication, implant, biomaterials, CT segmentation, FEA, simulation, patient’s specific

## Abstract

Additive manufacturing (AM) allows the creation of customized designs for various medical devices, such as implants, casts, and splints. Amongst other AM technologies, fused filament fabrication (FFF) facilitates the production of intricate geometries that are often unattainable through conventional methods like subtractive manufacturing. This study aimed to develop a methodology for substituting a pathological talus bone with a personalized one created using additive manufacturing. The process involved generating a numerical parametric solid model of the specific anatomical region using computed tomography (CT) scans of the corresponding healthy organ from the patient. The healthy talus served as a mirrored template to replace the defective one. Structural simulation of the model through finite element analysis (FEA) helped compare and select different materials to identify the most suitable one for the replacement bone. The implant was then produced using FFF technology. The developed procedure yielded commendable results. The models maintained high geometric accuracy, while significantly reducing the computational time. PEEK emerged as the optimal material for bone replacement among the considered options and several specimens of talus were successfully printed.

## 1. Introduction

In recent decades, AM processes have seen widespread exploration across various sectors like aeronautics, the automotive industry, and the biomedical industry. AM relies on layer-by-layer deposition techniques, enabling the creation of intricate geometries while minimizing waste. It is particularly well-suited for producing prototypes and custom functional parts tailored to individual patients. The utilization of AM techniques for developing and fabricating implants has been extensively researched. Many medical conditions necessitate the insertion of orthopedic implants. Implants come in diverse shapes, sizes, and materials. However, it is widely acknowledged that traditional implants (mass-produced items conforming to set standards) often fail to precisely match the unique anatomy of each patient [1,2,3,4,5]. An implant that does not fit properly can lead to sub-optimal results, compromising the patient’s healing process and the durability of the implant within the body. This is why there has been a growing interest in patient-specific implants over the past decade. Tailored precisely to match an individual’s anatomy, these implants aim to alleviate pain, reduce stress, and minimize the time required for the patient to adapt to the implant [1,2].

While the feasibility of producing patient-specific implants for reconstructive surgery, including orbital and craniofacial procedures, has been successfully demonstrated [3,6,7], challenges arise when considering load-bearing and joint locations such as the hip, knee, ankle, and foot. These areas are subjected to substantial stress and loads, presenting more complex requirements for implant design and durability. Consequently, developing patient-specific implants for these regions necessitates meticulous attention to biomechanical factors, material selection, and manufacturing techniques, to ensure optimal performance and longevity under demanding physiological conditions [3,4,5].

Despite these inherent challenges, significant strides have been made in the realm of patient-specific orthopedic implants. Numerous studies have focused on tailoring implants based on CT scans [2,8], while others have investigated optimal materials and 3D printing techniques for their production [9,10]. Notably, Hafez [11] successfully produced a universal talus implant using innovative topological optimization methods, showcasing the potential of advanced design strategies. Similarly, Snekhalatha’s development of a patient-specific hip prosthesis via finite analysis underscored the critical role of computational modeling in implant customization [12]. These efforts highlight the ongoing evolution of orthopedic implant technology, heralding promising advancements for improving patient outcomes and care.

**Figure 1:**
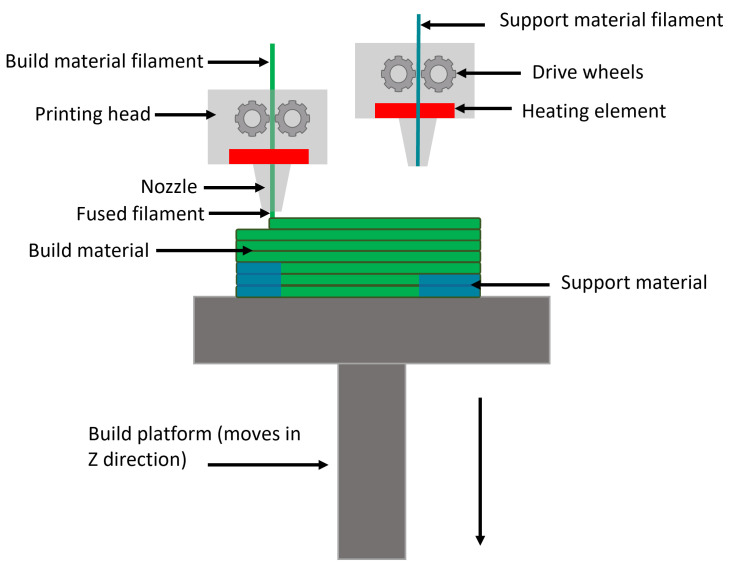
Schematic illustration of FFF process.

Of the various 3D printing methods available, fused filament fabrication (FFF) stands out as the most popular. Its popularity stems from its user-friendly nature, extensive material options, and notably, the affordability of the printing equipment, which is often compact enough to fit on a small desktop [13,14]. The FFF process is illustrated in Figure 1. In the FFF method, a thermoplastic or metallic filament (mixture of metallic particles and wax) undergoes continuous feeding into a heated chamber, where it melts. This molten material is then extruded through a nozzle and deposited layer-by-layer onto a heated table, adhering to a predefined pattern. The intended geometry is constructed along the Z-axis, as successive layers of melted filament are precisely laid down [13,14,15]. Thermoplastic filaments like poly(lactic acid) (PLA) or poly(ether ether ketone) (PEEK) stand as the primary materials utilized in FFF, respectively, for common and medical applications [13,15,16,17]. However, in recent times, metallic filaments such as stainless steel, titanium, and aluminum alloys have gained increasing traction, owing to their diverse applications across various fields [18,19,20].

Orthopedic implant manufacturing using fused filament fabrication (FFF) technology incorporates a variety of materials, encompassing both polymers and metallic alloys, selected based on their comprehensive mechanical properties, including strength, wear resistance, corrosion resistance, and proven bio-compatibility. Bio-compatibility is a pivotal parameter in implant development, delineating the interactions between the implant and the host environment. It is important to note that bio-compatibility is not universally standardized and can vary based on the implant’s location and the host’s response. Assessing a biomaterial’s bio-compatibility for a specific application involves conducting various tests, aiming for minimal host response across specific criteria: toxicity, mutagenicity, carcinogenicity, and immunogenicity [21].

Polymers play a crucial role in orthopedic implant fabrication due to their biocompatibility, versatility, and ease of processing with FFF technology. Among polymers, PEEK is recognized for its exceptional mechanical properties, high temperature resistance, chemical resistance, and excellent strength [17]. This material is often used in demanding applications where these qualities are necessary, such as aerospace, automotive applications, medical implants, and various industrial uses. Its properties make it particularly suitable for metal replacement [13]. According to a compilation of studies as summarized by Toth [22], PEEK demonstrates biocompatibility for bone implants and presents additional osteocompatibility. This signifies that a PEEK implant could potentially stimulate osteoblast production around the implant. Notably, PEEK showcases no chemical interactions with the host body, thereby preventing the release of ions or constituents into the host system [23].

In addition to polymers, metallic alloys play a vital role in orthopedic implant fabrication, particularly for load-bearing applications (notably hip prosthesis) requiring high mechanical strength and durability. Stainless steel, including alloys like 316L and 17-4 PH, offers excellent corrosion resistance, mechanical properties, and biocompatibility, providing long-term stability within the host body. The 17-4 PH stainless steel alloy filament is a multipurpose steel that can be used in FFF technology. It can be heat treated to reach a hardness of 36 HRc and polished for a better surface roughness. [19,24,25]. The 316L stainless steel filament is commercially designated as Ultrafuse 316L. It is one of the most common materials used in medical applications. It belongs to the family of austenitic stainless steels and offers excellent corrosion resistance, which is crucial for medical devices that come into contact with bodily fluids. [18,20,21,26]. Titanium and its alloys, such as Ti-6Al-4V and Ti-6Al-7Nb, combine low density and high bio-compatibility due to excellent corrosion resistance, making them a choice material for orthopedic implants [27,28], notably for hip and knee prostheses [29], dental implants [30], and craniofacial implants [31]. Like steel-based implants, titanium implants can also enhance cell proliferation through the layering of specific coatings [28,32].

The primary objective of this study was to create a numerical solid model aimed at identifying the most suitable materials for a talus implant through simulation. Once the geometry had been generated and simplified, the optimal material (among a predefined selection) was determined based on simulation results, and it was manufactured using FFF technology. For certain materials, post-processing is required to sinter the final model or remove critical support structures. In the subsequent phase of the analysis, various materials underwent physical testing.

## 2. Materials and Methods

### 2.1. Materials

In this comparison study, four specific materials—PEEK (Intamsys, Shanghai, China), 17-4PH stainless steel (Markforged, Waltham, MA, USA), 316L stainless steel (BASF), and Ti6Al4V (TVF)—were selected as potential candidates for fabrication of the implant. The mechanical properties of the materials are provided in Table 1.

### 2.2. Scanning and Mesh Generation

Accurate anatomical 3D models can be generated using numerical methods applied to volumetric image datasets. These datasets comprise volumetric pixels, commonly referred to as voxels. CT scans, in particular, are highly effective in capturing internal geometries, encompassing bones, organs, and even skin.

#### 2.2.1. CT Scan

CT scan images are employed to generate 3D surfaces. However, standard CT scans may lack the precision necessary for accurate 3D printing and simulations. The resolution of CT scans is pivotal in determining the accuracy of the resulting model and should align with the required precision for additive manufacturing (AM). Key parameters that influence segmentation quality include slice thickness, slice spacing, and pixel size.

Slice thickness represents the portion of a patient’s anatomy captured within one slice, while slice spacing denotes the gap between adjacent slices. Pixel size determines the 2D precision within each slice. Numerous studies have emphasized the importance of these parameters in achieving optimal segmentation quality [37,38]. For optimal results, the slice thickness should be less than 1.25 mm, ideally paired with a slice spacing equal to or smaller than the slice thickness. Additionally, the pixel size should be under 0.6 mm to ensure high-quality segmentation [39].

While many segmentation programs allow post-scan reduction in pixel size, it is important to note that this primarily aids in segmentation rather than enhancing the scan’s precision. When determining the field of view (FOV) for the scan—meaning the area encompassed within—it is crucial to include the necessary anatomy for segmentation while minimizing the scope, to reduce the patient’s exposure to radiation [37,39,40].

Adjusting the mentioned parameters according to the size of the anatomy being segmented is essential. Thin bones, for instance, may require an exceptionally low slice thickness to ensure accurate representation [37]. For larger or less intricate anatomies, a slice thickness greater than 1.25 mm may suffice. CT protocols are responsible for defining and adapting these parameters based on specific applications and imaging requirements [38,40].

In this study, the healthy talus of a patient was under consideration. The CT scan parameters for the foot included a slice thickness of 1 mm, a slice spacing of 1 mm, and a pixel size of 0.59 mm. The segmentation of foot bones was executed using 3DSlicer 4.11 software, employing the ‘thresholding’ and ‘grow from seeds’ methods. The ‘thresholding’ method quickly categorizes pixels into foreground and background based on their gray levels, making this faster with lower CPU requirements, suitable for simple visual observation. On the other hand, the ‘grow from seeds’ method enables simultaneous segmentation of different anatomies with higher precision and less manual intervention.

Typically, the segmented anatomy is saved as STL or OBJ files, as these formats can be directly utilized in additive manufacturing processes. Additionally, a STEP file is prepared specifically for simulation purposes.

#### 2.2.2. Remesh of Generated Surfaces and Solid Generation

The large STL files generated from scans require remeshing before integration into computer aided design (CAD) or simulation software. Software limitations, particularly in managing the number of surfaces and the computational load associated with a high count of mesh elements, necessitate a remeshing process. This step addresses imperfections like spikes and ensures a smoothed surface. A smooth surface is crucial for resolving contacts in numerical simulations and eliminating stress concentrations. Remeshing the bones and improving surface smoothness contributes to result convergence, significantly reducing the computational time in simulations.

However, it is crucial to acknowledge that remeshing surfaces can lead to a loss of geometrical information. To evaluate when this loss became critical, a large STL file underwent remeshing with various percentages of reduction. The original mesh (before any reduction) was then compared with each of the reduced meshes. Using the original mesh as a reference, the software randomly selected around 50,000 points within the mesh and endeavored to find their equivalent points in the reduced mesh. An iterative process was employed to minimize the distance between each pair of points.

The following criteria were then extracted: average distance, standard deviation, and mean distance. The average distance was calculated by summing all distances between pairs of points from the two meshes and dividing the total by the number of pairs. Standard deviation indicates the dispersion of distances around the average value (a low standard deviation value means distances are closely grouped around the average). The mean distance was subsequently calculated by extrapolating the sample results to the entire mesh, instead of the initially considered 50,000 points. Consequently, the lower the values of average distance, mean distance, and standard deviation, the higher level of geometric accuracy. From the analysis of these three criteria, a critical threshold of reduction was determined.

### 2.3. Simulation

#### 2.3.1. Modelization of the Ankle

Bone is a mineralized connective tissue comprising two structures: cortical (dense) and trabecular (spongy) bone. Cortical bone primarily forms the outer layer of bones, while trabecular tissue is situated within the metaphysis, epiphysis, and medullary cavity at the ends of long bones [41]. It is also present in short bones [41]. These connective tissues differ significantly in their organization, resulting in distinct mechanical behaviors. Cortical bone, characterized by its density, showcases a high compressive strength, playing a prominent role in bone mechanics. Conversely, trabecular bone features a honeycomb-like structure optimized for facilitating fluid exchange and absorbing shocks [42]. These mechanical disparities were incorporated into the simulation.

**Figure 2:**
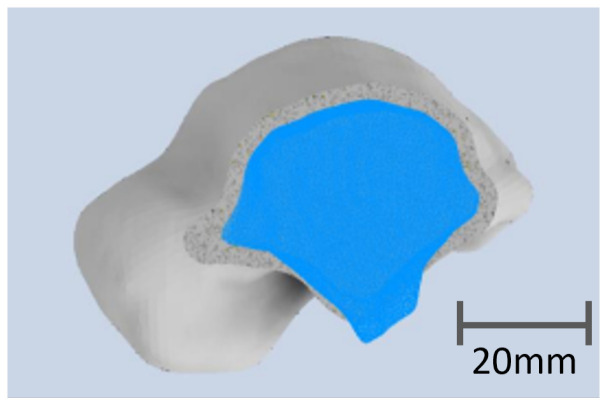
Transversal view of the numerical talus with the cortical bone in gray defined by a thickness of 2 mm and the trabecular bone in blue.

The numerical talus comprised two distinct solids, with the outer layer representing the cortical bone. Typically, the thickness of cortical bone ranges between 2 and 3 mm, varying significantly based on the bone’s location and the patient’s medical condition, as indicated in the literature [43,44]. For the considered CT scan, the thickness of the cortical bone was estimated at 2.8 mm, which is consistent with the literature. In the simulation and print model, the thickness was set to 2 mm, to place our analysis in the most conservative geometry. The second solid, nested within the first, modeled the trabecular bone, as depicted in the accompanying figure (see Figure 2). To mimic the porous nature of this tissue, the second solid was assigned a reduced percentage of the elasticity modulus compared to the outer shell.

The simulation aimed to determine the material most similar to the original bone. Both the cortical and trabecular solids underwent changes in mechanical properties. The material properties for each type are outlined in the Table 2.

**Figure 3:**
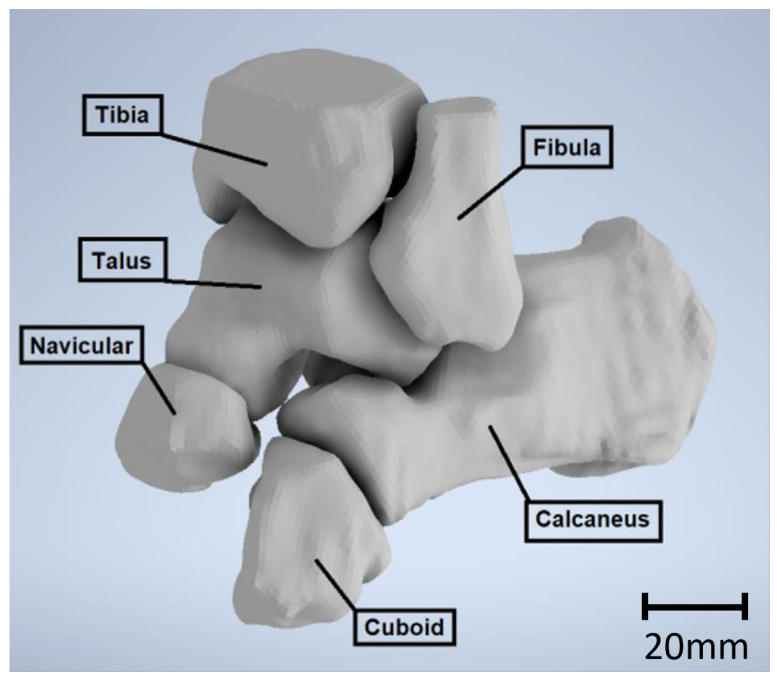
Solid model of the ankle.

Beyond the talus, all bones in direct contact were replicated in the simulation, as depicted in the accompanying figure (see Figure 3). Ligaments, varying in size, thickness, and rigidity, serve as connectors between bones. In the simulation, springs were selected to replicate these ligaments, due to their defined characteristics of length and rigidity, enduring tensile and compression loads. The stiffness of these ligaments was previously documented and summarized by Ramlee et al. [47]. Table 3 provides the ligaments used in our simulation with their respective stiffnesses.

**Figure 4:**
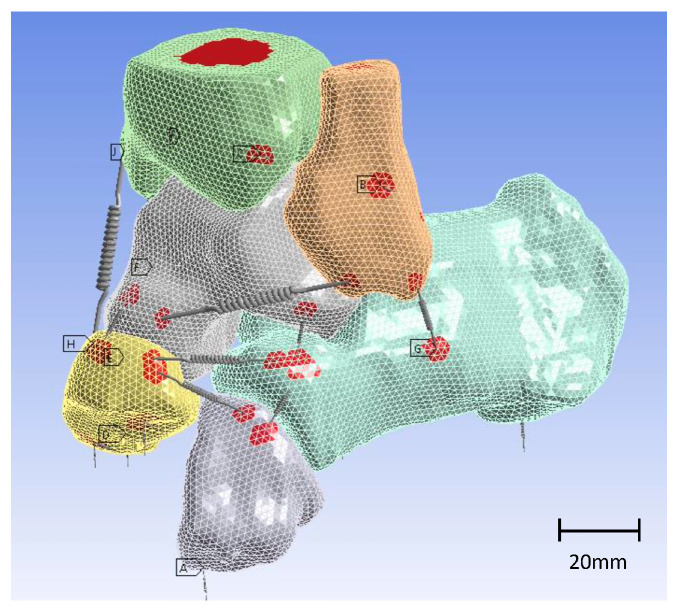
Three-dimensional model of the ankle with springs as ligaments. Remote points are highlighted in red.

To replicate larger areas of connection between ligament and bone and prevent stress concentration at boundary conditions with imposed displacements and in regions where the foot contacts the ground, remote points were implemented in the simulation model, as illustrated in Figure 4.

Tendons and muscles were omitted from the current finite element analysis (FEA) due to their patient-specific behavior, making it challenging to establish standardized comparisons. However, cartilage, crucial for damping between bones, was accounted for in the simulation. Utilizing frictional contact allowed the replication of this effect, set at a coefficient of 0.05 for contact between the bones. This same coefficient was applied uniformly across all considered materials. Consequently, the comparison of results focused solely on the modification of material behavior. By fully replicating the ankle’s articulation and subsequently mirroring it, the simulation comprehensively modeled the joint mechanics.

**Figure 5:**
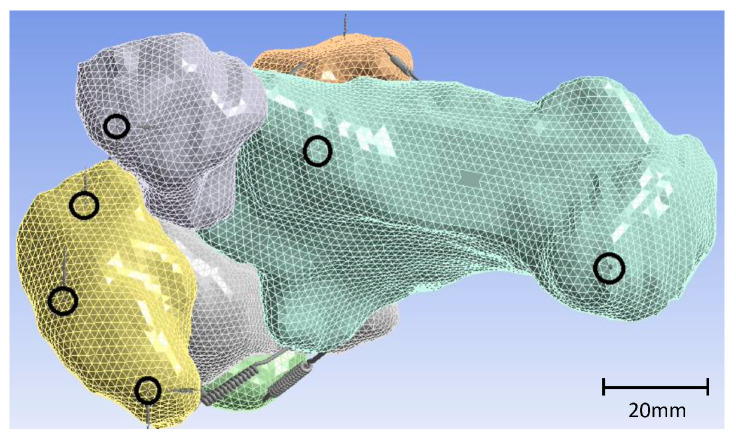
Fixation points of strings connected to navicular, cuboid, and calcaneus, achieving grounding of the model.

The entire model was anchored to the ground through clamping. The foot was secured to the ground using springs in three directions, while the other bones were interconnected using general contacts. The stiffness of the springs was carefully determined to ensure the stability of the model in both longitudinal and torsional directions. Specifically, the springs were connected to the navicular, cuboid, and calcaneus bones, as illustrated in Figure 5.

#### 2.3.2. Loads and Boundary Conditions

The loads exerted on the ankle primarily affect the tibia and fibula, influenced by the body’s upright stance. These loads are contingent upon the patient’s body weight [48]. The maximum loads on bones are typically attributed to muscle action. Several studies have focused on the loads endured by the bones during numerous activities. For instance, when a healthy individual hops on one leg, the force generated can be approximately 3.5 times their body weight [48]. Loads applied on the lower limbs during walking and jogging reach on average 5 times and 8 times the body weight, respectively, according to Bergmann et al. [49] and Glitsch et al. [50]. During high-impact activities like jumping or running, such loads can increase up to 15 times the body weight [51].

**Figure 6:**
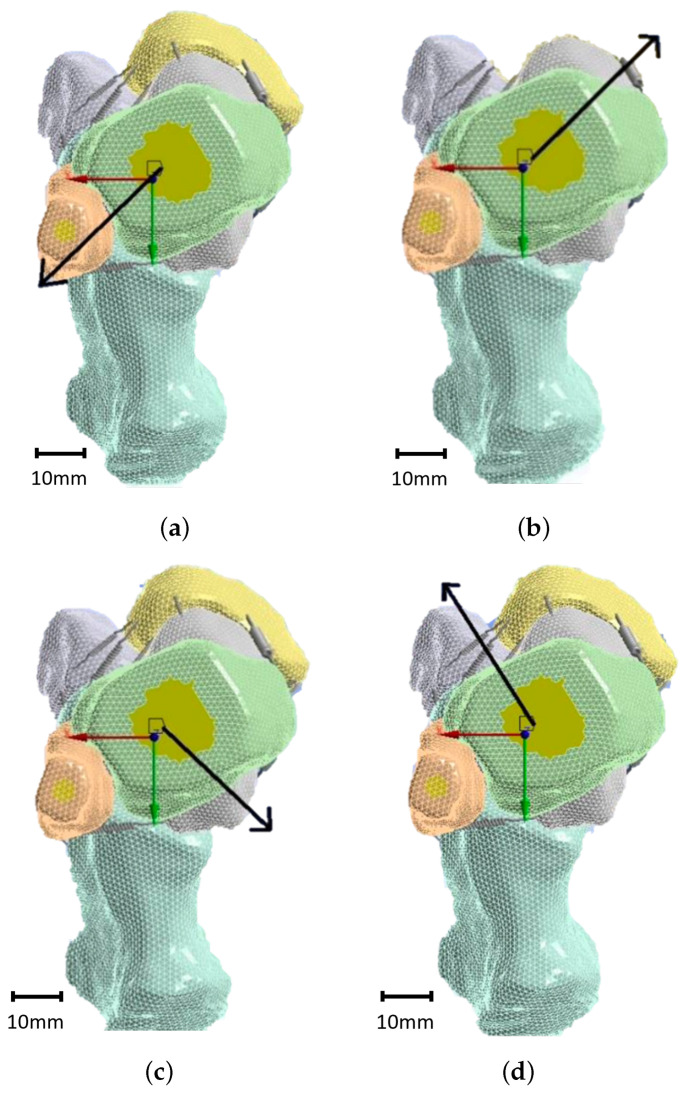
Load cases considered in the present simulation: ILB (**a**), ELF (**b**), ELB (**c**), and ILF (**d**).

The forces acting on the tibia and fibula were directed in different ways to simulate various movements. Pronation involves a combination of eversion and dorsiflexion, which leads to an interior lateral backward (ILB) movement. This was represented by load case 1, as illustrated in Figure 6a. Conversely, exterior lateral forward (ELF) movement represents an inversion and plantarflexion simultaneously, known as supination and depicted by load case 2, as shown in Figure 6b. The exterior lateral backward (ELB) movement represents a combination of eversion and plantarflexion, depicted by load case 3, as visible in Figure 6c. Lastly, the interior lateral forward (ILF) movement is a combination of inversion and dorsiflexion, simulated by load case 4, as described in Figure 6d. Displacements in the X, Y, and Z directions for each case are summarized in Table 4. The finite element model comprised several sources of non-linearity, including multiple contacts and large displacements. Attempting to drive the model via force led to numerous convergence issues in achieving a stable solution. To address these convergence issues and attain a solution, the model was instead driven by displacements. This approach enabled the attainment of converged solutions, while significantly reducing the solving time. The reaction forces were derived from the imposed displacements, which are detailed in Table 4.

### 2.4. Three-Dimensional Printed Prototype Implant

The shell thickness of manufactured implants plays a pivotal role in determining their mechanical properties, resembling the cortical bone structure. To ascertain the desired thickness of the produced implants, print tests were conducted. Boxes measuring 40 × 40-mm were printed with varying values of wall line count. The shell thickness was theoretically calculated by multiplying the wall line count by the nozzle diameter. The influence of printing parameters, such as layer thickness, the number of wall lines for the shell, and the percentage of infill, was measured on reference parts. This comparison was conducted using both PLA and PEEK materials. The number of wall lines directly affected the thickness of the shell. Additionally, a comparison was made of the infill pattern and wall thickness using two different slicer software programs: Cura v5.7.0 and Intamsuite v3.8. Table A1 presents a summary of the specific printing parameters used in the print tests.

## 3. Results

### 3.1. Results of the Segmentation

**Figure 7:**
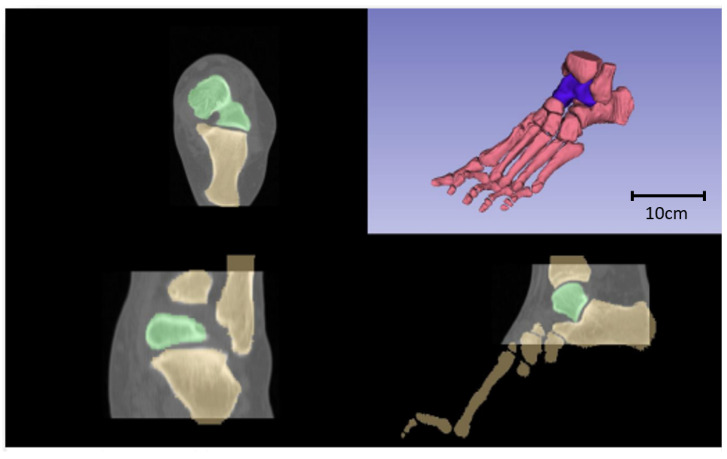
Axial (upper left quadrant), frontal (lower left quadrant), sagittal (upper right quadrant), and isometric (upper right quadrant) views of the resulting segmentation, with the talus highlighted in green and purple, respectively.

Only bones were segmented, and Figure 7 displays various transversal views of the segmented bones, emphasizing the talus in green. Furthermore, an isometric view of the joint is presented in the upper right quadrant, with the talus marked in purple.

**Figure 8:**
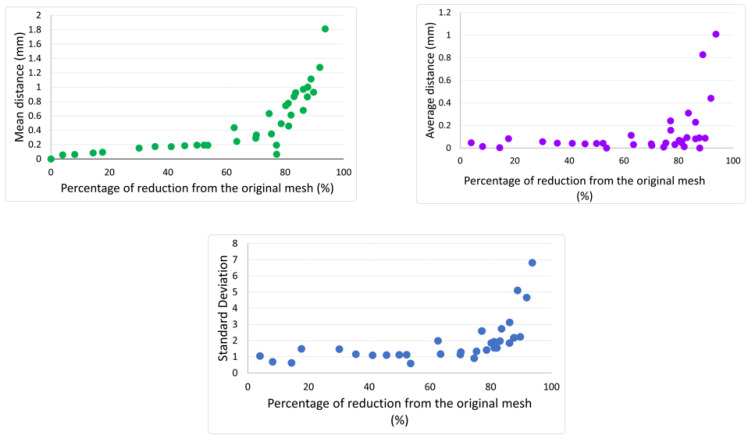
Influence of mesh reduction on the geometrical accuracy of the original talus.

**Figure 9:**
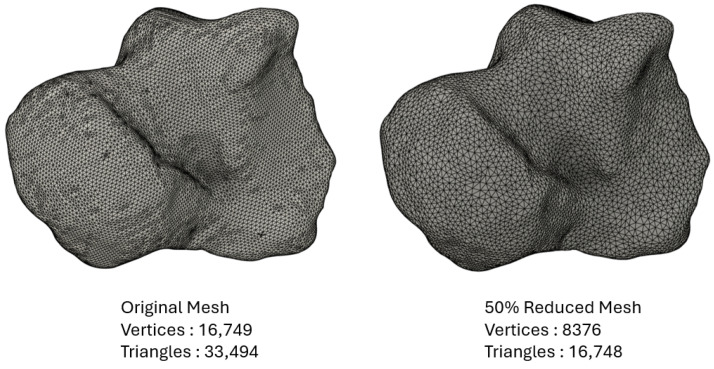
Original mesh of the talus and the 50%-reduced version used in the simulation.

Following the methodology outlined in Section 2.2, a comparison between the original talus model and the various remeshed versions (with differing percentages of mesh reduction) was conducted. The evolution in the three criteria—average distance, mean distance, and standard deviation—with the percentage of mesh reduction is displayed in Figure 8. Below 60% of mesh reduction, the values of average distance and mean distance were below 0.2 and the standard deviation was lower that 2. Between 60% and 80% of mesh reduction, the values of the three criteria rose slightly to 0.25, 0.5, and 3, respectively. These values were still acceptable, thus the impact of the remesh on the geometrical accuracy remained limited. However, starting at 80% reduction, the values of the three criteria underwent an exponential increase. Therefore, the critical threshold was determined to be 80%. For this specific study, the mesh reduction was maintained below 50% to guarantee optimal geometrical accuracy. The original mesh of the talus and the 50%-reduced version (which was used in the simulation) are displayed in Figure 9.

### 3.2. Simulation Results

The successful modeling of ankle articulation enabled a comparison between various materials (PEEK, stainless steel, and titanium) and the original bone. After an unsuccessful attempt at force-driven simulation, the model was instead driven by displacements, as detailed in Table 4. This displacement-based approach facilitated the attainment of converged solutions for each of the four movements considered in the analysis. Reaction forces in the X, Y, and Z directions, as well as the total reaction force, were computed and compared to the corresponding values for the bone. Table 5 only presents the percentage differences between the considered material and the bone, while detailed values of the reaction forces can be found in Appendix A.

According to the computed reaction forces, PEEK exhibited the lowest difference compared to the bones, with a maximum of 24% for the 50% infill. Decreasing the infill of PEEK resulted in higher but still acceptable difference values. In contrast, stainless steel and titanium showed much higher differences, reaching values up to 200%. Stainless steel and titanium were excessively rigid compared to the behavior of the original bone material.

The magnitude of the reaction forces in the displacement-driven model corresponds to 14 times the body weight of a standard patient, equating to a safety factor of 4, for the load condition of a patient standing on one leg. This safety factor is rational, especially in scenarios where a person experiences a sudden fall from a certain height without any damping.

**Figure 10:**
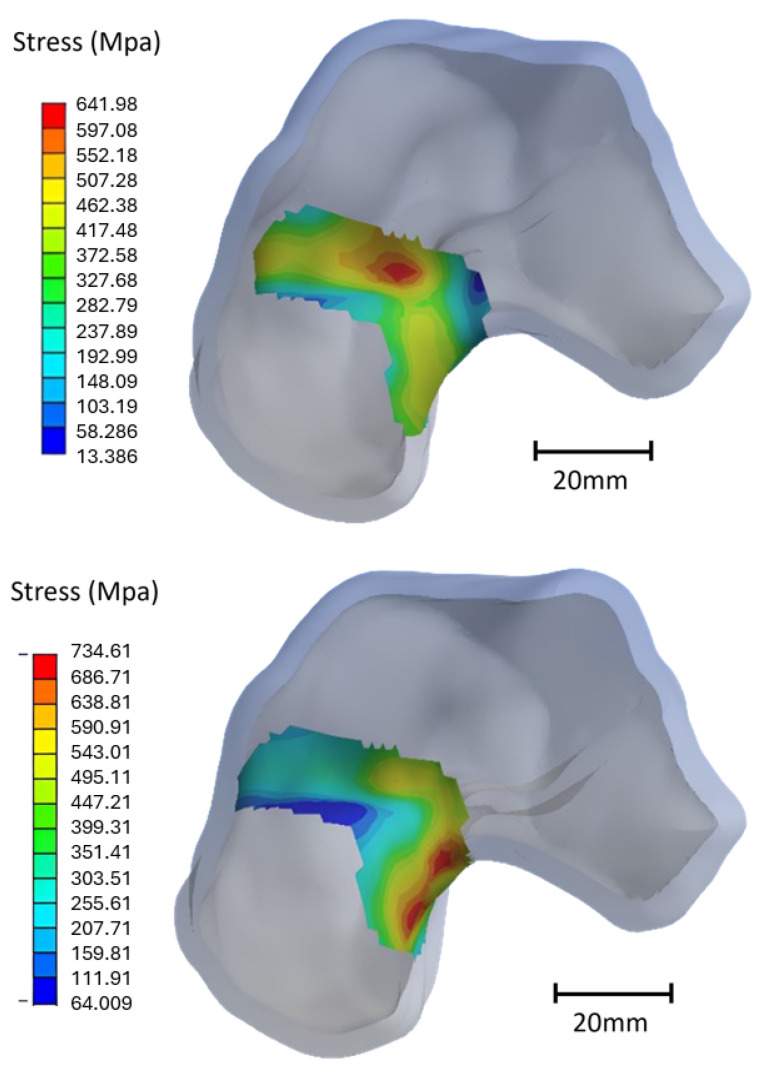
Stress concentration areas localized at the top and bottom of the neck, respectively, for the exterior lateral forward movement.

Fractures of the talus primarily occur at a specific location known as the neck of the talus [52]. In alignment with this, the simulation results also revealed that the highest strains and stresses were concentrated in the neck of the talus, indicating a potential risk of fracture in this area. Equivalent strains and stresses were calculated around the neck, highlighting two distinct stress concentration zones, one at the top and another at the bottom, as illustrated in Figure 10. The values of stress localized in the talus neck are presented in Table 6.

Based on the stress distribution, two worst-case postures were identified: ELF and ELB. Consequently, the simulation focused specifically on these two cases. PEEK demonstrated significantly lower stress magnitudes compared to the original bone material, being less than half. In contrast, the stress in the talus models made of titanium and stainless steel was comparable to that of the bone.

It is noteworthy that the stress magnitudes for all materials surpassed their respective tensile strength, forming the basis for comparing the behavior of different materials. To address this issue and prevent excessively high stress magnitudes, material models should incorporate non-linearity, including some plasticity. The assumption is that a force equivalent to 14 times the weight of a patient can potentially lead to a fracture of the talus neck. The linear material model resulted in high stress magnitudes, and attention should also be given to the elastic strains.

**Figure 11:**
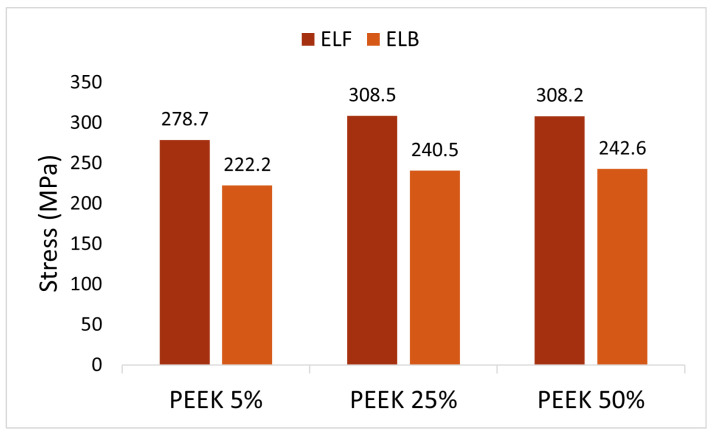
Equivalent Von Mises stress (MPa) in the neck of the talus for different infills of PEEK implant for the worst-case scenarios (ELF and ELB).

Equivalent stress and strain in the neck of the talus were calculated for PEEK models with 5%, 25%, and 50% infill of the inner solid, to determine the optimal configuration. The Young’s modulus of fully dense PEEK was reported as 3950 MPa [16]. For PEEK models with lower infill percentages, the Young’s modulus was obtained by multiplying the Young’s modulus of fully dense PEEK by the percentage of infill [16]. Consequently, PEEK models with 5%, 25%, and 50% infill exhibited Young’s moduli of 197.5 MPa, 987.5 MPa, and 1975 MPa, respectively. The resulting Von Mises stresses are illustrated in Figure 11. No significant difference between infill percentages was observed. The 5% PEEK infill showed slightly lower values compared to the other infills, although they were comparable. Additionally, the infill did not influence the location of the maximum stress values.

**Figure 12:**
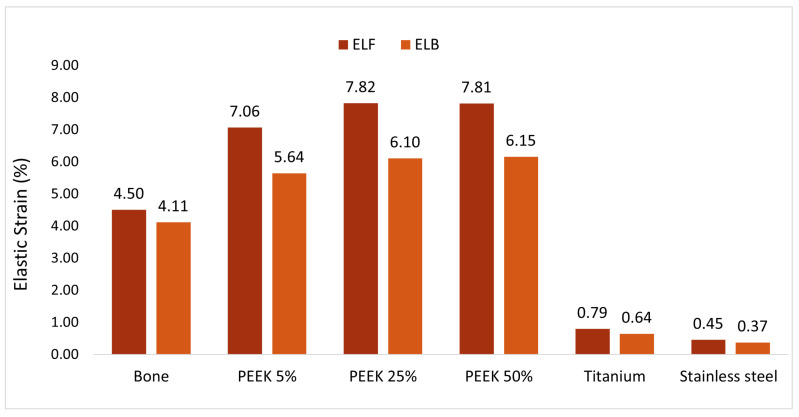
Elastic strain of the different materials for the two worst-case scenarios in percentage.

Elastic strain is a crucial consideration for implant assessment. The computed elastic strains of the various materials are presented for the two worst-case scenarios in Figure 12. Focusing solely on the strain, it was indicated that a fracture of the talus under a force equivalent to 14 times the weight of the patient is unlikely. PEEK consistently exhibited values closest to those of natural bone, whereas titanium and stainless steel demonstrated a brittle behavior with an elastic strain below 0.5%. PEEK showcased a high level of flexibility, even surpassing that of bone. The computed elastic strains of PEEK remained far below the acceptable elongation at break provided by the material supplier. However, analyzing the elastic strain of PEEK for various infill values revealed no significant trend.

Consequently, PEEK emerged as a potentially suitable material for a talus implant. However, the percentage of infill remains a critical variable to determine. The stress and strain analysis did not reveal any trends regarding the optimal value of infill, but according to the computed reaction forces, a talus with 50% infill behaved similarly to the natural bone material.

### 3.3. Print of the Talus Implant

**Figure 13:**
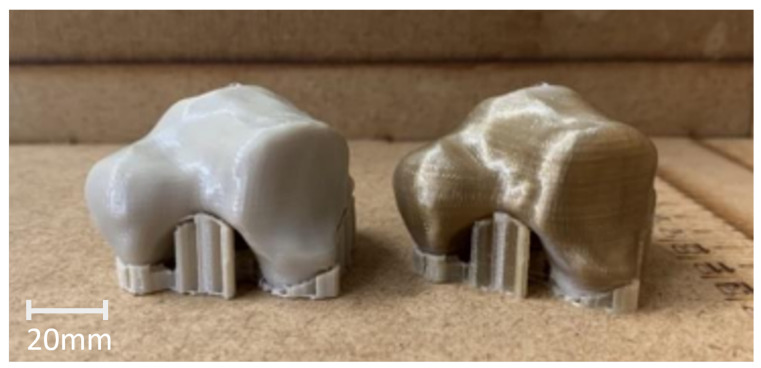
PEEK talus after the heat treatment (**left**) and before (**right**).

Multiple specimens were 3D printed in PEEK, each with varying percentages of infill. In Figure 13, two specimens are presented before and after heat treatment. Despite stainless steel not emerging as the optimal replacement material based on the simulation results, a few specimens were printed for evaluation. Detailed parameters of the models, including their mass, are provided in Table 7. Mass is a critical parameter in implant development, and it should ideally align with the mass of the original bone.

According to the literature, the mass of the human talus is approximately 20 g, considering factors such as the sex, age, height, and other health considerations of the patient [53]. PEEK implants with 50% infill exhibited a mass of 20.5 g, the closest value compared to the original talus. PEEK models with lower infill percentages also maintained a mass close to that of the original bone. In contrast, implants based on stainless steel weighed around 100 g, a significant deviation from the corresponding bone mass. Since titanium did not yield satisfactory results in the simulation and is expensive to print, no titanium implants were printed for weight comparison. Given the parameters selected in this study, stainless-steel-based implants seem to be the least optimal choice for talus replacement.

Taking into account the computed reaction forces, stress, and strain results, and mass, a talus with 50% infill behaved similarly to natural bone material, making it the preferred choice for the implant material.

## 4. Discussion

The segmentation and simplification procedure developed for generating 3D solid numerical models has demonstrated excellent results in terms of precision and efficiency. These models are created with high accuracy and simplified to reduce the mesh size, thus decreasing the computational time without significant loss of geometric detail. Studies have shown that various smoothing algorithms, such as Laplacian smoothing, can effectively reduce mesh complexity, while preserving critical geometric features [38,54]. The precision of these models also allows them to be successfully 3D printed. Specifically, the implants made from PEEK with a 50% infill closely resembled the original bone structure.

Optimizing the implant surface post-treatment is crucial to enhance the relative movement of the ankle and reduce friction between the bones. This refinement aims to facilitate ligament attachment to the implant. Tailoring surface roughness is also vital for promoting cell adhesion and proliferation. Several techniques can be employed, such as sandblasting, which has been shown to enhance surface roughness, thereby improving osteoconduction, cell adhesion, bone-to-implant contact, and removal torque. These benefits support the viability of sandblasted PEEK as a bone implant [55,56]. Another promising technique for optimizing the surface roughness of polymeric implants is plasma coating. Studies by Wang et al. [57] and Han et al. [58] demonstrated that plasma coating significantly enhanced cell adhesion and proliferation. A further advancement would be to customize the surface treatment to achieve varied roughnesses across different areas of the implant. For instance, applying a higher roughness to specific regions could promote cell adhesion, while maintaining a smoother surface at the interface between the implant and adjacent bones could reduce friction and improve overall functionality.

Research suggests that antibiotic coatings are also critical for the integration of implants within the patient’s body [59,60]. Silver nanoparticles, used by Deng et al. [61] as a coating agent on PEEK implant surfaces, were proven to prevent biofilm formation and limit immune reactions, while maintaining the mechanical properties of the coated implant. Alternatively, totarol, known for its strong antibacterial properties, was successfully applied to PEEK implants, leading to reduced immune response at the implant location [62].

The simulation model produced satisfactory results in terms of strains and contact pressure for comparative purposes. Moreover, other studies have demonstrated similar and satisfactory results using analogous procedures. Shim et al. [63] developed a hybrid method capable of generating patient-specific FE meshes from sparse or incomplete clinical datasets, though their focus was on a single bone rather than an articulation. Varghese et al. [64] developed FE models based on CT scans that accurately determined strain and stress responses to two different loading conditions. The simulation model developed in this study incorporated four different types of movements combining bending and torsion. Validation of the model generation under more complex loading conditions will be pursued for greater clinical relevance.

However, it is important to note that certain damping effects, such as those from tendons, synovial fluid, blood vessels, or skin, were not considered in the current model for simplification reasons. While these complexities could influence the absolute output results, as highlighted by Viceconti et al. [65], the comparison study remains valid, since the same simplifications were applied uniformly across all models, thereby damping them consistently [54].

The linear material model employed may not accurately reflect real-world behavior, and was used for comparison purposes, although the calculated strain values remained below the ultimate strain. The PEEK implant was predicted to withstand 14 times the patient’s weight without failure. For those concerned with result accuracy, implementing a nonlinear material law could provide more precise stress and strain values, albeit at the expense of convergence stability and increased computational cost. Additionally, incorporating the non-linearity of large displacement options could enhance the result accuracy.

The friction coefficient, set at 0.05 based on the literature, did not undergo thorough estimation of its influence on results. This coefficient is influenced by various factors such as the age, sex, and health conditions of the patient [66], making it difficult to determine accurately [67]. Moreover, even with an accurate friction coefficient between the bones implemented in the simulation, the friction coefficient between the implant and the surrounding bones may differ, as highlighted by Rancourt et al. [68]. Therefore, additional investigations should be conducted to examine the influence of the friction coefficient on the overall mechanical behavior of the ankle joint following the implementation of the implant.

The mechanical properties of the considered materials were also derived from the literature, but performing physical tensile and fatigue tests on the printed specimen would contribute to more accurate results and instill confidence in the simulation model.

Considering the long-term behavior of the printed model, particularly the adhesion between different layers of 3D-printed implants, is critical. Ongoing physical tests will be instrumental in further validating the simulation model [59].

## 5. Conclusions

In summary, the simulation model, derived from a CAD model generated from CT scans, can be considered conservative. Notably, the PEEK implant with 50% infill closely resembled the original bone characteristics, showcasing a capacity to withstand forces equivalent to 14 times the patient’s body weight. Furthermore, the successful production of implants via 3D printing, especially the 50% PEEK implant exhibiting a weight comparable to that of natural bone, confirmed its suitability for clinical use. Nevertheless, further mechanical testing is needed to fully confirm and validate the performance and effectiveness of the patient-specific implant.

## Figures and Tables

**Table 1:** Mechanical properties of selected materials.

Material	Young’s Modulus (MPa)	Poisson Ratio	Ultimate Strength (MPa)	Yield Strength (MPa)	Elongation at Break (%)
PEEK [33]	3950	0.39	114	80	9.1
316L stainless steel [34,35]	200,000	0.28	734	586	53
17-4PH stainless steel [35,36]	191,000	0.28	1230	1050	13
Ti6Al4V [27,32]	110,000	0.31	897	828	16

**Table 2:** Mechanical properties of cortical and trabecular bones [45,46].

Material	Young’s Modulus (MPa)	Poisson Ratio	Ultimate Strength (MPa)	Yield Strength (MPa)	Elongation at Break (%)
Cortical Bone	16,350	0.3	150	115	2
Trabecular Bone	155	0.2	66	51	50

**Table 3:** Stiffness of the ligaments represented in the model [47].

Connected Bones	Stiffness (N/mm^2^)
Fibula-Calcaneus	125
Fibula-Talus	101
Fibula-Talus 2	78
Tibia-Talus	80
Tibia-Navicular	40
Tibia-Calcaneus	122
Talus-Navicular	70
Cuboid-NAvicular	70
Calcaneus-Cuboid	70
Navicular-Talus	70
Navicular-Calcaneus	70
Calcaneus-Talus	70
Calcaneus-Navicular	70

**Table 4:** Imposed displacements in FEA.

Displacement	Time Step (s)	Value in X Direction (mm)	Value in Y Direction (mm)	Value in Z Direction (mm)
	1	0	0	−2.5
ILB	2	5	0	−2.5
	3	5	5	−2.5
	1	0	0	−2.5
ELF	2	−5	0	−2.5
	3	−5	−5	−2.5
	1	0	0	−2.5
ELB	2	−5	0	−2.5
	3	−5	5	−2.5
	1	0	0	−2.5
ILF	2	5	0	−2.5
	3	5	−5	−2.5

**Table 5:** Reaction force difference from bone in percentage.

Movement	Time Step (s)	PEEK 5% Infill	PEEK 25% Infill	PEEK 50% Infill	Stainless Steel	Titanium
	1	48.7	17.3	−5.3	−190.5	−176.5
ILB	2	46.9	17.5	−1.9	−144.3	−134.4
	3	44.8	11.8	−9.4	−134.6	−126.8
	1	48.7	17.3	−5.3	−190.5	−176.5
ELF	2	45.1	8.8	−17.0	−200.6	−186.6
	3	46.7	7.5	−16.7	−179.0	−167.3
	1	48.7	17.3	−5.3	−190.5	−176.5
ELB	2	45.1	8.8	−17.0	−200.6	−186.6
	3	42.0	1.6	−24.0	−170.9	−160.3
	1	48.7	17.3	−5.3	−190.5	−176.5
ILF	2	47.0	17.5	−1.9	−144.3	−134.4
	3	46.2	11.2	−11.7	−169.3	−158.6

**Table 6:** Equivalent stress in the neck of the talus (MPa).

Material	ILF	ILB	ELF	ELB
Bone (MPa)	521.8	642.0	734.6	672.2
PEEK (MPa)	192.9	191.9	308.5	240.5
Titanium (MPa)	572.3	486.5	878.7	703.2
Stainless steel (MPa)	597.4	509.1	918.3	737.1

**Table 7:** Printing parameters and mass of the produced implants.

Model	Material	Layer Height (mm)	Wall Line Count	Shell Thickness (mm)	Top and Bottom Thickness (mm)	Infill (%)	Mass (g)
A	PEEK	0.1	5	1.86	1.2	25	14.4
B	PEEK	0.1	5	1.86	2	50	20.5
C	PEEK	0.1	5	1.86	2	25	16
D	PEEK	0.1	5	1.86	2	5	12.4
E	316L	0.1	3	1.8	2	25	95.6
F	17-4PH	0.125	8	2	2	25	102.7

## Data Availability

The original contributions presented in the study are included in the article, further inquiries can be directed to the corresponding authors.

## Abbreviations

The following abbreviations are used in this manuscript:

AM	Additive manufacturing
FFF	Fused filament fabrication
CT	Computational tomography
FOV	Field of view
PLA	Polylactic acid
PEEK	Polyetheretherketone
CAD	Computer aided design
FEA	Finite element analysis
ILB	Interior lateral backward
ELF	Exterior lateral forward
ELB	Exterior lateral backward
ILF	Interior lateral forward

## Appendix A

**Table A1:** Printing parameters.

	PEEK	PLA	316L	17-4PH
Nozzle size	0.15 mm	0.15 mm	0.4 mm	0.45 mm
Layer height	0.1 mm	0.1 mm	0.1 mm	0.125 mm
Print temperature	400–440 °C	200–230 °C	245 °C	220 °C
Print speed	25 mm/s	70 mm/s	30 mm/s	25 mm/s
Heatbed	180 °C	50–60 °C	90–105 °C	115 °C
Fan speed	50%	50–100%		
Drying temperature and time	150 °C during 4 h	-	-	-
Required moisture content	below 200 ppm	-	-	-
Post treatment	Annealing 150 °C/1 h, 200 °C/2 h, 150 °C/30 min	-	Catalytic debinding 10 h, sintering under H2 environment	Debinding into Opteon SF-79, annealing 1040 °C/1 h, 550 °C/4 h.

**Table A2:** Details of the print tests.

Printer	Parameters from the Slicing						Measurements of the Produced Parts		
	Material	Shell Thickness (mm)	Layer Thickness (mm)	Wall Line Count	Infill (%)	Nozzle Diameter (mm)	Shell Thickness (mm)	Bottom Thickness (mm)	Ratio
Funmat HT	PEEK	2	0.1	5	25	0.4	1.86	2.01	0.37
	PEEK	2	0.1	6	25	0.4	2.30	2.02	0.38
Prusa i3 MK3	PLA	2	0.1	5	25	0.4	2.10	2.01	0.43

**Table A3:** Reaction forces for ILB movement in (N).

	**Bone**				**PEEK5**				**PEEK25**			
Time	Force in X	Force in Y	Force in Z	Total Force	Force in X	Force in Y	Force in Z	Total Force	Force in X	Force in Y	Force in Z	Total Force
1	673.7	−200	−21,145	22,156	328.2	221.5	−11,358	11,365	326.1	61.1	−18,325	18,328
2	9595.6	31.2	−27,082	28,732	5862.6	503.5	−14,603	15,244	8067.7	−135.4	−22,283	23,699
3	8656.4	16,247	−35,614	40,163	5511.1	9944.5	−19,033	22,170	6612.1	15,356	−31,231	35,424
	**PEEK50**				**Stainless Steel**				**Titanium**			
Time	Force in X	Force in Y	Force in Z	Total Force	Force in X	Force in Y	Force in Z	Total Force	Force in X	Force in Y	Force in Z	Total Force
1	321.7	−192.9	−23,319	23,322	−244.5	−3439.2	−64,280	64,373	−132.4	−3149.9	−61,173	61,254
2	9376.2	−560.9	−27,717	29,266	18,374	−1595.1	−67,718	70,184	17,752	−1555.9	−64,941	67,341
3	7399.9	18,782	−39,036	43,947	14,427	37,915	−85,062	94,240	13,828	36,816	−82,172	91,098

**Table A4:** Reaction forces for ELF movement in (N).

	**Bone**				**PEEK5**				**PEEK25**			
Time	Force in X	Force in Y	Force in Z	Total Force	Force in X	Force in Y	Force in Z	Total Force	Force in X	Force in Y	Force in Z	Total Force
1	673.7	−200	−22,145	22,156	328.2	221.5	−11,358	11,365	326.1	61.1	−18,325	18,328
2	−7756.7	607.4	−26,144	27,277	−5287.8	−521.2	−14,000	14,974	−7482.7	−31.9	−23,720	24,873
3	−7731.7	−10,242	−33,525	35,897	−6002.9	−7228.5	−17,899	20,216	−8084.1	−9851.1	−30,660	33,203
	**PEEK50**				**Stainless Steel**				**Titanium**			
Time	Force in X	Force in Y	Force in Z	Total Force	Force in X	Force in Y	Force in Z	Total Force	Force in X	Force in Y	Force in Z	Total Force
1	321.7	−192.9	−23,319	23,322	−244.5	−3439.2	−64,280	64,373	−132.4	−3149.9	−61,173	61,254
2	−8955.4	264.4	−30,620	31,904	−19,042	174.8	−79,741	81,983	−18,276	198.2	−76,011	78,177
3	−9346.6	−11,854	−39,129	41,940	−16,798	−20,332	−96,625	100,160	−16,215	−20,210	−92,369	95,935

**Table A5:** Reaction forces for ELB movement in (N).

	Bone				PEEK5				PEEK25			
Time	Force in X	Force in Y	Force in Z	Total Force	Force in X	Force in Y	Force in Z	Total Force	Force in X	Force in Y	Force in Z	Total Force
1	673.7	−200	−22,145	22,156	328.2	221.5	−11,358	11,365	326.1	61.1	−18,325	18,328
2	−7756.7	607.4	−26,144	27,277	−5287.5	521.2	−14,000	14,974	−7482.7	−31.9	−23,720	24,873
3	−7599.2	18,210	−36,826	41,780	−4855.5	11,414	−20,839	24,251	−7870.6	19,766	−35,203	41,133
	**PEEK50**				**Stainless Steel**				**Titanium**			
Time	Force in X	Force in Y	Force in Z	Total Force	Force in X	Force in Y	Force in Z	Total Force	Force in X	Force in Y	Force in Z	Total Force
1	321.7	−192.9	−23,319	23,322	−244.5	−3439.2	−64,280	64,273	−132.4	−3149.9	−61,173	61,254
2	−8955.4	264.4	−30,620	31,904	−19,042	174.8	−79,741	81,983	−18,276	198.2	−76,011	78,177
3	−9859.1	24,377	−44,637	51,807	−20,595	473,862	100,680	113,160	−19,718	45,879	−96,628	108,770

**Table A6:** Reaction forces for ILF movement in (N).

	**Bone**				**PEEK5**				**PEEK25**			
Time	Force in X	Force in Y	Force in Z	Total Force	Force in X	Force in Y	Force in Z	Total Force	Force in X	Force in Y	Force in Z	Total Force
1	673.7	−200	−22,145	22,156	328.2	221.5	−11,358	11,365	326.1	61.1	−18,325	18,328
2	9596.6	31.2	−27,082	28,732	5862.6	503.5	−14,063	15,244	8067.7	−135.4	−22,283	23,699
3	3715.3	−2639	−29,806	30,153	2326.7	−3019.9	−15,769	16,223	2639.8	−4495.4	−26,269	26,781
	**PEEK50**				**Stainless Steel**				**Titanium**			
Time	Force in X	Force in Y	Force in Z	Total Force	Force in X	Force in Y	Force in Z	Total Force	Force in X	Force in Y	Force in Z	Total Force
1	321.7	−192.9	−23,319	23,322	−244.5	−3439.2	−64,280	64,373	−132.4	−3149.9	−61,173	61,254
2	9376.2	−560.9	−27,717	29,266	18,374	−1595.1	−67,718	70,184	17,752	−1555.9	−64,941	67,341
3	2784.5	−4603.8	−33,241	33,673	5381.1	−11,631	−80,179	81,196	5235.7	−11,093	−77,104	77,985
